# A multi-component intervention to reduce bias during family planning visits: qualitative insights on implementation from Burkina Faso, Pakistan and Tanzania

**DOI:** 10.1186/s40834-024-00296-6

**Published:** 2024-07-09

**Authors:** Corrina Moucheraud, Alexandra Wollum, Muhammad Ali Awan, William H. Dow, Willa Friedman, Jean-Louis Koulidiati, Amon Sabasaba, Manisha Shah, Zachary Wagner

**Affiliations:** 1 Department of Public Health Policy and Management, New York University School of Global Public Health, New York, NY USA; 2 Department of Community Health Sciences, University of California Los Angeles Fielding School of Public Health, Los Angeles, CA USA; 3 APPNA Institute of Public Health, Jinnah Sindh Medical University, Karachi, Pakistan; 4 Department of Health Policy and Management, University of California Berkeley School of Public Health, Berkeley, CA USA; 5 Department of Economics, University of Houston, Houston, TX USA; 6 Institut Supérieur des Sciences de la Santé, Université Nazi Boni, Bobo‑Dioulasso, Burkina Faso; 7 Health for A Prosperous Nation (H-PON), Dar es salaam, Tanzania; 8 University of California Berkeley Goldman School of Public Policy, Berkeley, CA USA; 9 RAND Corporation, Santa Monica, CA USA

**Keywords:** Contraception, Qualitative research, Implementation science, Intervention, Burkina Faso, Pakistan, Tanzania

## Abstract

Beyond Bias was an intervention introduced in Burkina Faso, Pakistan and Tanzania, with the aim of reducing health worker bias toward young, unmarried and nulliparous women seeking family planning services. This study used qualitative methods – based on interviews with health workers who participated in the intervention, managers at health facilities that participated in the intervention, and policy and program stakeholders at the national level – to understand implementation experiences with the intervention. The results offer insights for organizations or countries seeking to implement Beyond Bias or similar programs, and point to some other key implementation challenges for multi-component interventions in lower-resource settings. The intervention, developed using a human-centered design approach, was seen as key for successful implementation but there were logistical challenges. The digital intervention was disruptive and distracting to many. In addition, the non-financial rewards intervention was perceived as complex, and some participants expressed feeling discouraged when they did not receive a reward. Beyond Bias did not sufficiently attend to the “outer setting,” and this was perceived as a major implementation barrier as it limited individuals’ capacity to fully achieve the desired behavior change; for example, space constraints meant that some health facilities could not ensure private services for all clients. There were scalability concerns related to cost, and there is uncertainty whether diversity of contexts (within and across countries) might constrain implementation of Beyond Bias at scale.

## Introduction

Health workers’ attitudes may be an important determinant of the services they provide: if health workers hold biased attitudes toward a certain group, they may provide different care to this group [1, 2]. An example of this dynamic is health worker bias toward different clients seeking family planning services [3, 4], which may result in a range in adverse outcomes – for example, clients being counseled on fewer method choices, being denied services, or facing unreasonable barriers to care (like requiring consent from other parties) [5–11]. Groups commonly receiving this differential care include young people, unmarried people, and those without children [4]. In addition to resulting in worse-quality care [12], differential treatment may impact future care-seeking among those discriminated against [11, 13]. Changing health worker attitudes and reducing biases may therefore be an important intermediate goal toward improving quality of care for groups that suffer discrimination during encounters with the health system.

Beyond Bias is one of very few interventions to date that directly targets health worker bias in family planning service provision [4, 14–16]. In a randomized controlled trial, the intervention was found to effectively change biased attitudes and beliefs and improve quality of care for young women [14, 17]. These results have generated broad enthusiasm for scaling-up the intervention to other settings and institutionalizing the intervention through collaborations with Ministries of Health. Here we describe implementation experiences with the intervention, which had several characteristics making it amenable to an implementation evaluation: it was a multi-component intervention with standardized content, developed via a human-centered design approach, and delivered across three different countries (in Burkina Faso, Pakistan, and Tanzania). This paper will provide valuable insights for organizations considering implementing the Beyond Bias intervention, and generalizable recommendations about the implementation of multi-component interventions in low-resource settings.

## Methods

This was a three-country qualitative study, involving in-depth interviews with health workers (people who are primarily responsible for direct family planning service provision) and facility managers (people whose job includes or is entirely managerial) at sites that implemented the Beyond Bias intervention (as this analysis focuses on implementation experiences), and with national-level policy and program stakeholders.

### Description of the intervention

Beyond Bias was a multi-component, provider-facing intervention aimed at improving family planning care by reducing health worker bias toward young, unmarried, and nulliparous women seeking these services. Beyond Bias aimed to increase the provision of non-judgmental, empathetic, unbiased family planning care to young (aged 15–24), unmarried, and nulliparous clients, and ultimately give these women access to a full range of appropriate contraceptive methods. The intervention package was developed through a human-centered design process that lasted over two years and included extensive formative research (a segmentation analysis to understand “types” of providers and profiles of their biases, and a literature review), an iterative process of intervention design with multiple rounds of prototyping, and a pilot at 227 health facilities in the three study countries (for more details about the intervention design process, see [16]). The intervention ultimately comprised three activities.

First, there was an in-person, highly interactive meeting called “*Summit*,” which was held once (at the beginning of the intervention period) and educated health worker attendees about bias during family planning care. Summit used personal stories from young people to stimulate guided discussion among health worker attendees about their own experiences with bias, and attendees were encouraged to commit to reducing bias in their practice. The goal was to activate workers’ empathy for the needs of young people, and to create action plans for reducing their own bias.

Second, there was a forum called “*Connect*” for moderated discussion about bias and family planning care. Connect aimed to increase knowledge, and peer support, about family planning care for young people; this was achieved through sharing of case studies, tips from technical experts to address misunderstandings and misinformation, creation of a “safe space” for sharing experiences and challenges with peers, and review of goals to maintain motivation and commitment. In Burkina Faso, Connect was delivered through in-person meetings at health facilities; in Pakistan, Connect was delivered via WhatsApp; and in Tanzania, there were both facility meetings and WhatsApp content. There was weekly Connect engagement for 8–10 weeks in each country (an intensive phase), followed by monthly engagement for the remainder of the 12-month intervention period (a continuous learning phase).

Third, there were non-monetary performance-based quarterly awards called “*Rewards*.” Each quarter, facilities with the lowest and most-reduced levels of biased care (as measured using data from client exit surveys and defined according to the six principles of unbiased care developed for this study: (1) safe, welcoming space; (2) sensitive communication; (3) simple, comprehensive counseling; (4) seek understanding and agreement; (5) say yes to a safe method, and (6) security of information) were recognized publicly at a ceremony, and received small tokens such as a certificate. Rewards aimed to motivate health workers to participate in the intervention and maintain the provision of unbiased services, and to create accountability for service quality.

### Study setting

The Beyond Bias intervention was implemented by Pathfinder International in three countries: Burkina Faso, Pakistan and Tanzania. These countries were selected due to their high prevalence of unmet need for contraception among young women [18]. Characteristics of the participating countries are shown in Table 1. In Tanzania, there were 73 public-sector clinics in Dar es Salaam that participated; in Burkina Faso, 78 public clinics in the regions of Centre, Haut-Bassins, and Cascades; and in Pakistan, 80 private clinics in Karachi. Facilities were randomized 1:1 into the intervention or standard of care arm. The intervention was launched in September 2020 and concluded in September 2021.

**Table 1:** Characteristics of participating countries

	Burkina Faso	Pakistan	Tanzania
Global region & income group [19]	Africa; Low-income economy	South Asia; Lower-middle income economy	Africa; Lower-middle income economy
Female population aged 15–49 years, 2021 [20]	5.20 million	57.25 million	15.31 million
GDP per capita (PPP, constant 2017 I$), 2021 [21]	$2,180	$5,232	$2,582
Total fertility rate, 2021 [20]	4.77 births per woman	3.47 births per woman	4.73 births per woman
Adolescent fertility rate, 2021 [20]	110.5 births per 1000 women ages 15–19	42.3 births per 1000 women ages 15–19	123.7 births per 1000 women ages 15–19
Any modern method use among women aged 15–49, 2021 [22]	30.4%	28.1%	38.5%
Median age at first sexual intercourse (women aged 25–49), data year as noted	17.7 (2010) [23]	20.7 (2017-18) [24]	17.2 (2015-16) [25]

### Overview of evaluation design

The Beyond Bias intervention was evaluated using a randomized controlled trial design, to evaluate impacts on attitudes and beliefs of health workers; client-centered care; family planning methods dispensed; and client perceptions of treatment by health workers. For more information about the impact evaluation and all data sources, see [14]. The evaluation collected data from multiple sources, including the qualitative data that were used for this analysis. These qualitative data were collected through in-depth interviews with health workers, health facility managers, and national-level key policy and program stakeholders.

### Interview guide

There were three separate interview guides developed, one per type of interviewee. The guide for health workers captured experiences with and opinions about the intervention activities, including what worked well and what did not. The guide for health facility managers similarly asked about the intervention activities, plus included questions about perceived value of the intervention and impressions of potential scalability. The guide for policy/program stakeholders was focused on opinions about intervention implementation and potential scalability. The guides were translated from English to local languages by professional translators at local research firms.

### Site and participant selection

Health workers and facility managers were sampled by selecting health facilities, and then inviting respondents within these. First, within each country, facilities that participated in the Beyond Bias intervention were stratified by geographic sub-region; responsiveness to the intervention (based on their level of improvement in bias indicators between the first two quarters of the intervention period); urban versus suburban location (in Burkina Faso and Tanzania); and volume of youth clients served (based on exit surveys conducted for the evaluation). We purposively selected facilities to maximize variation along these parameters: 11 facilities in Burkina Faso, 22 facilities in Pakistan, and 11 facilities in Tanzania (see Table 2). (As each facility in Pakistan only had 1 provider, we included a greater number of facilities in the qualitative assessment in order to attain the target health worker sample size in each country.) At each selected facility in Burkina Faso and Tanzania, 2 randomly-selected health workers were invited to participate in an interview, and at each selected facility in Pakistan, all health workers were invited to participate. In addition, at 5 selected facilities in Burkina Faso, the head nurse at the facility (“Infirmier chef de poste”) was invited to participate in an interview, and at 5 selected facilities in Tanzania, the head of the Reproductive Health Department was invited to participate.

**Table 2:** Characteristics of participants in the Beyond Bias study, and of the qualitative study sample

	Burkina Faso	Pakistan	Tanzania
**Beyond Bias intervention participation**			
Number of facilities that participated in the Beyond Bias intervention & study	78	76	73
Health facility sector	Public	Private	Public
Providers per facility (average)	11.1	1.0	4.3
Family planning clients per month per facility (average)	101.7	21.1	226.5
**Qualitative study sample**			
Number of facilities that participated in qualitative data collection activities	11	22	11
Number of health workers interviewed	21	21	21
Number of managers interviewed	5	0 (not applicable)	5
Number of policy/program stakeholders interviewed	13	16	9

Members of the project Advisory Committee in each country were invited to participate in interviews, as policy and program stakeholders. These Committees were comprised of national and subnational government officials, project staff, technical directors from the implementing organization, representatives from local and global non-governmental organizations, local researchers, and local health workers and administrators.

### Data collection

At intervention endline, a researcher called each selected respondent (health worker, manager, or policy/program stakeholder) to invite them to participate; some program/policy stakeholders also received an email invitation. Those who expressed willingness were then scheduled for an interview at a convenient time for them; if the selected health worker was not interested or could not be contacted after 4 attempts, we replaced them with another respondent from the same or a similar facility. Interviews with health workers and managers were held in-person at a private location in the health facility, and interviews with policy/program stakeholders were held in-person at a location of their choosing or via phone.

Trained qualitative researchers from the in-country research teams led each interview. Interviews were conducted in Swahili in Tanzania, Urdu and English in Pakistan, and in French and other local languages in Burkina Faso. All respondents gave oral informed consent to participate in an interview, and provided permission to audio record the interview. Health workers and managers were given refreshments to thank them for participating in the interview. Data were collected between July and August 2021.

### Data analysis

The interview audio recordings were transcribed and translated to English. Any identifying information (names, ages, dates) was removed. We iteratively generated a codebook based on the interview guides and following a review of the transcripts. We double-coded a set of interviews from each country, within each interview type, to reach consistency in code applications. We coded transcripts using Dedoose software. Thematic analysis was conducted to understand experiences and implementation of the intervention by country, and themes were compared across countries where possible. We used constructs from theoretical frameworks – the Consolidated Framework for Implementation Research (CFIR) for use in low- and middle-income countries [26]) for the health worker and manager interviews, and the ExpandNet framework [27] for policy/program stakeholder interviews – to identify themes related to implementation and scale-up. The CFIR is a widely-used implementation science framework that characterizes factors associated with implementation in the domains of: characteristics of the intervention, outer setting, inner setting, characteristics of individuals, and processes. ExpandNet is a framework used to describe what factors may be associated with potential scalability of an intervention, in the domains of: the innovation, the resource team, and the user organization. Here we present first findings related to intervention implementation (using the CFIR), and then findings about scalability (using ExpandNet).

### Ethical review

We obtained ethics approval to conduct this study from RAND’s Human Subjects Protection Committee (IRB00000051), and we obtained approval from ethical review boards in all three countries: the Comité d’Ethique pour la Recherche en Santé IRB in Burkina Faso (IRB00013418), the Research and Development Solutions Institutional Review Board in Pakistan (IRB00010843), and the National Institute for Medical Research in Tanzania (IRB00002514). All respondents provided informed consent to participate, and all research was conducted in accordance with the Declaration of Helsinki.

## Results

We interviewed 21 health workers in each country; plus 5 managers in each of Burkina Faso and Tanzania; and 13 policy/program stakeholders in Burkina Faso, 16 in Pakistan, and 9 in Tanzania. (The total qualitative sample was therefore comprised of 111 interviews: 39 respondents in Burkina Faso, 37 in Pakistan, and 35 in Tanzania.) (See Table 2.) Thematic results are presented aligned with the main domains of CFIR and ExpandNet.

### Implementation: co-created intervention yields satisfied participants, but challenges remain (CFIR innovation domain)

In all three countries, health workers and managers were largely very satisfied with the intervention. They spoke about how much it had changed their behavior toward young, unmarried and nulliparous people seeking family planning services (*relative advantage*). This was attributed to increased knowledge and to changes in attitudes and self-efficacy. As one health worker in Pakistan said: “*I used to be a bit timid before. Now I fear no one. Before everyone looked at me like I was a sinner but now I have proof that this thing [family planning services] is also good from a medical point of view.*” A health worker in Tanzania shared: “*You made me realize that a young client is important to the community and me. Therefore, I need to give her the best service at the right time. It also reminded me that I need to prioritize young people to help them achieve their goals.*” A health worker in Burkina Faso said, “*The [Beyond Bias] project has created an awakening within our service and in our practices. It has brought a change in our practice. Now we welcome all clients without prejudice, without any problem, it is open to all.*”

The *innovation design* was seen as unique and appealing. Respondents felt that the multi-component design was helpful, as the different activities were complementary and built upon one another. There was a lot of enthusiasm for Summit in all three countries, particularly its interactive format; in the words of a health worker from Tanzania: “*Other [trainings], when you go, you hold a notebook and pen, but at Summit you will play, you will sing*.”

Connect was well-liked in Burkina Faso and Tanzania, where it created communities of practice and a sense of shared responsibility to act (“*When we do these sessions, the actions we take are collective. Together we decide what to do and everyone follows what the group decides*” [Burkina Faso]; “*It becomes difficult to do it alone, but when you are in a group like this, you help one another in doing this, by sharing ideas*” [Tanzania]). This was explained also as being important for gaining buy-in within the health facility: “*When we do these sessions, the actions we take are collective. Together we decide what to do and everyone follows what the group decides*” (Burkina Faso); “*[These] meetings at the health facility are very important because you discuss the challenges that arise while providing services… and you come up with solutions. The sessions were very good and they helped us see what to do to improve where there was a challenge*” (Tanzania).

The digital format of Connect also cultivated a sense of community for some: “*All the providers, we are all talking like a family. I think the act of learning and teaching is never completed. No one can say that I am 100% perfect. We are continuously learning things. When we hear the experience of others, we come to know that there are still most of things we haven’t know*” (Pakistan); “*You find yourself sharing ideas with different people that you do not know. Maybe someone from [the city of] Kigamboni has encountered some challenges there, he comes and shares in the group… this person suggests this [a solution], another one suggests this, and maybe if you have thoughts you give some ideas. So you find yourself gaining from different places, so even if you didn’t know something before, you get to know it*” (Tanzania)—but it also posed challenges. In Pakistan, the WhatsApp-based functionality was often described as frustrating and disruptive: “*Sometimes the continuous sharing of content and ringing of the phone cause disturbance. Like some girls [other health workers in the intervention] shared unnecessary information like memes or pictures, so because of that, we sometimes do not read the important messages because we think they are not important too.*” In Pakistan, where the participating health facilities were often operated out of health workers’ homes, some people spoke about how the Connect messages were disruptive to their work/life balance: “*I can’t check Connect regularly as I am busy with household chores*.” Challenges with Connect were also mentioned by some respondents in Tanzania who lacked sufficient connectivity: “*There are some that have normal phones, very few have these internet phones.*”

There was a range of reactions to Rewards. Some people in all countries – including those who did and did not receive a reward – found it motivating. Representing this sentiment, one health worker at a lower-performing site in Tanzania said: “*You see your colleagues take the award, you look at what you did wrong and this enables us to do better and better*.” But many health workers and managers found it frustrating and disheartening, especially when they did not understand the data or calculation for who received a reward: “*Obviously, I get jealous. I am also working hard, why I haven’t gotten good marks? I don’t understand*” (Pakistan); “*We are struggling to get a prize, because we go and see our peers get a prize but we are empty-handed. It hurts so much*” (Tanzania).

There were differing opinions about the *innovation source*. In Pakistan, many respondents lauded the implementing organization and saw its staff as instrumental to the success of the intervention. In Tanzania, several people said they wish the Ministry of Health or health workers had been more involved in designing the intervention.

### Implementation: contextual challenges were a substantial barrier (CFIR outer setting domain)

In Pakistan and Tanzania, some respondents spoke about the challenges posed by community context (*local attitudes*), and in particular counseling quality being hindered by low knowledge/awareness by women seeking services (“*Sometimes it is difficult to give counseling… as they are illiterate. Some understand but some do not*” [Pakistan]), and social norms about care-seeking (“*In our community, people still don’t accept that youth need family planning. They think that if we teach them, it is like telling them ‘go and do’ [have sex]… Even parents or relatives or guardians, they still think is not correct to have family planning at a young age*” [Tanzania]). The role of social norms and lack of awareness around family planning was mentioned repeatedly as a barrier to family planning services during interviews in Pakistan.

Especially in Burkina Faso and Tanzania, health workers and managers spoke about implementation challenges related to the health system (*local conditions*, *financing*). The most common health system constraints were lack of space, health worker shortages, and, in Burkina Faso, stockouts of family planning commodities.

Due to severe lack of space, it is challenging to provide privacy during family planning visits; some respondents, like this health worker in Burkina Faso, saw this as problematic for young people seeking care: “*If we could have a space just for the FP…it would be very helpful… There are young people who don’t want to come and meet their parents*.” The intervention also taught about the importance of privacy for offering unbiased care, and participants were frustrated about their inability to execute this action.

Insufficient staff was perceived to impact implementation in a few ways. First, there was a lot of staff turnover so new people joined the facility but had not received training (“*New workers, when they come, we try to explain to them and they participate in Connect, but we feel that their way of doing things is not the same as for those who participated in Summit*” [Burkina Faso]); and those who were trained left (“*The attendant that we had in the beginning for Beyond Bias was transferred from our facility… Our energy decreased because a big percentage didn’t know about Summit*” [Tanzania]). Additionally, lack of staff limited participants’ ability to engage in the intervention: “*During the Connect sessions, if you are the only one providing the service, you want to follow the Connect but … you cannot follow because you are busy doing something else in the delivery room*” (Burkina Faso). Interestingly, health workers in Pakistan also spoke about demands on their time, but more commonly attributed this to lengthier counseling due to the intervention (“*When FP patients take more time, other patients get irritated*”) and to household or other personal duties (“*You go [to Summit] in the morning and come back in the evening… These events disturb our routine activities*”).

### Scale-up: desirable but costly (ExpandNet resource team domain) and may require changes (ExpandNet innovation domain)

There was overall enthusiasm about the intervention and its potential, and policy and programmatic stakeholders expressed largely positive impressions about the possibility of taking it to scale. However, in all three countries, these stakeholders expressed concern that the intervention is too expensive to take to scale (*resources*): “*We want to, but we don’t have the resources to scale it up. We have the infrastructure, but the not necessary funds to do it*” (Pakistan).

In Burkina Faso and Tanzania, respondents also mentioned wanting to see more involvement of diverse partners, and integration into government activities (*credible*). In the words of a policy/program stakeholder from Burkina Faso: “*We can’t continue as a country to rely only on projects. I don’t think that anyone else should work on a project. We have to work to make it a program. And for it to be a program, it has to be integrated into what we are doing*.” Participants commonly expressed it was as critical for the government to lead any scale-up of the intervention.

In Pakistan and Tanzania, several policy and program stakeholders mentioned wanting to see the impact evaluation results before deciding if the intervention should be taken to scale (*observable*): “*We are waiting for the impact data. Until those results, we can’t say that it is ready for implementation* (Pakistan). (It should be noted that these interviews were completed before the impact evaluation data were available.) In contrast, other stakeholders in these countries felt they had already seen data demonstrating the effectiveness of the intervention.

Respondents were also concerned that local factors would impact scale-up (*compatible*): “*The more we extend, the more we’ll have to deal with obstacles, either from a religious or cultural point of view*” (Burkina Faso); “*The regions are different, there are cultural issues and some are hard to change, they might not give you good results*” (Tanzania). This was also mentioned with respect to sub-national variation in infrastructure like internet access, mentioned in Tanzania (“*[For Connect], access to the internet is not very accessible in Tanzania, so some places will have limited access… so that can also be a barrier*”); and lack of security in certain communities of Burkina Faso (“*Well, coming back to what I said before, the challenges, well I say maybe the security challenges, it becomes a constraint*”).

Related to this, especially in Burkina Faso and Tanzania, people spoke about the importance of being able to modify the intervention: “*We continue learning from adaptations… It is like eating chips—one will add chilli and one will not add chilli, but the base is that two people have eaten chips. So, there can be an adaptation, it is allowed to happen*” (Tanzania). Adaptations were seen as important to fulfill differing facility-level needs, or to accommodate the local social/cultural context. Some policy/program stakeholder respondents in Tanzania felt that, due to the human-centered design process, the intervention package was locally-tailored – i.e., optimized for the Dar es Salaam setting – and therefore might not work with other populations, so adaptations would be critical.

## Discussion

The Beyond Bias intervention was effective at changing providers’ biased attitudes and beliefs and at improving quality of care [14]. In this qualitative assessment of implementation experiences among health workers and key programmatic and policy stakeholders involved in the Beyond Bias intervention, we identify perceived barriers and facilitators to implementation and scale-up that may offer insights for other multi-component, multi-country projects. The findings from the accompanying Beyond Bias impact evaluation are promising [14], so these qualitative findings about implementation suggest opportunities and challenges for scale-up.

First, these results suggest that a strong, co-developed intervention design may be an important ingredient for successful implementation. Despite contextual differences across the three settings, respondents endorsed the intervention in very similar ways: the complementarity of the different intervention activities was highly praised, as was the innovative interactive design. Beyond Bias was developed and refined through a two-year human-centered design process, and this engagement of end-users may have helped strengthen the intervention and ultimately eased its implementation. Public health programs and projects must recognize the critical role of intervention design, and the potential power of human-centered design, for successful implementation [28–30].

There were, however, intervention component-specific challenges during implementation that may offer some generalizable lessons. For example, many health workers found Rewards (the non-financial award activity) confusing or discouraging. Interventions that incorporate financial and non-financial incentives – such as conditional cash transfers, facility scorecards, peer comparison/benchmarking interventions –should seek to simplify and clarify their metrics, methods, and messaging. Such transparency, and more attention to the behavioral mechanisms (and potential for negative experiences), might strengthen the implementability of these initiatives.

In the countries that used a digital format for Connect (Pakistan and Tanzania), there were numerous implementation challenges. These offer important insights for other programs seeking to equip health workers with digital tools. There were technical challenges, ranging from the types of phones that health workers use, to lack of consistent network connectivity. There were practical issues, like not having enough time to monitor one’s mobile phone during the workday. Some people also found the phone alerts disruptive and annoying. Given the rise in health-related digital tools globally, including in low- and middle-income countries [31, 32], we need to better understand how end-users experience these interventions – and how their design can be tweaked to improve implementation.

Beyond Bias delivered its activities to health workers but did not modify the outer context, which is known to be an important potential implementation determinant but out of scope for this project. There were perceived health system constraints – lack of space, shortages of health workers, commodity stockouts – that health workers said limited their capacity to fully “translate” their changed attitudes into changed behaviors with clients. Likewise, many respondents (particularly in Pakistan) spoke about difficulties due to low awareness and acceptance in the community. Behavior change programs like Beyond Bias may wish to incorporate multi-level interventions, in order to address external factors that may limit implementation and may ultimately impact outcomes of the intervention. These results also indicate that outer setting factors – health system, communities and context – may also affect scalability. Interventions attending to multiple levels of the socioecological model have larger impact [33–35]. It is impossible for all programs to address all constraints, however, so implementers should carefully consider which contextual factors may be most essential to “bundle” with behavior change interventions in order to see maximum impact.

This assessment found many similarities in implementation experiences and scalability perceptions across these three different contexts, but other factors may be sources of variation. For example, the format of Connect drove its implementation: those who experienced in-person Connect felt that it created a sense of community and buy-in around the topic of bias, and while digital Connect created a helpful network, it also had numerous challenges (as described above). Additionally, there were differences in the types of facilities that participated in Beyond Bias across countries, and this may have affected implementation: respondents in Burkina Faso and Tanzania – where high-volume, public sector facilities participated – were much more likely to mention health system constraints like lack of space or health worker shortages; and in Pakistan, where the participants were sole-provider practices, respondents spoke about juggling intervention duties with their other work and household tasks. (It should be noted that the facilities that participated in Beyond Bias may not be fully representative in each country [sector, size, etc.], which may limit the generalizability of these findings.) Although many frameworks treat these as separate determinants, we encourage greater attention to understanding how the interplay between intervention design, inner context, and outer context affects implementation.

Several limitations of this analysis should be noted. First, respondents were not blinded to their intervention status, and may therefore have been overly enthusiastic about their implementation experiences during these interviews. We tried to minimize this by using a research team that had been uninvolved in the intervention, and by explaining the role of the independent evaluation to each respondent. However, we cannot rule out this possible social desirability bias. There may also have been recall bias, if respondents found it more challenging to report experiences with earlier aspects of the intervention. Lastly, different individuals may have had varying degrees of engagement with the intervention (which only ran for one year), and we were not able to explore whether implementation experiences or impressions differed according to an individual’s degree of exposure to the intervention.

## Conclusion

Family planning services are poor-quality in many countries [36, 37]. Certain groups – young people, unmarried people, and nulliparous people – may experience particularly poor care, and health worker bias about these groups may be one factor contributing to this disparity. The Beyond Bias intervention was one of very few projects to date that attempted to directly change health worker attitudes about these groups that commonly experience discrimination during family planning care. The multi-component, multi-country intervention encountered many implementation facilitators and barriers, which may offer lessons for other complex projects and programs. We also identify several areas deserving further exploration and study, including how incentive-based interventions can be strengthened, the importance of attending to contextual factors and of considering multi-level interventions, and challenges that may face digital interventions in low-resource settings.

## Data Availability

The data used in this study are not made available since some responses contain potentially identifiable information at multiple levels including for individuals, health facilities, and organizations. Sharing the data would breach compliance with the protocol approved by research ethics boards for this study.
